# Occurrence and Geographic Distribution of Plant-Parasitic Nematodes Associated with Citrus in Morocco and Their Interaction with Soil Patterns

**DOI:** 10.3390/life12050637

**Published:** 2022-04-25

**Authors:** Btissam Zoubi, Fouad Mokrini, Abdelfattah A. Dababat, Mohammed Amer, Cherki Ghoulam, Rachid Lahlali, Salah-Eddine Laasli, Khalid Khfif, Mustafa Imren, Oumaima Akachoud, Abderrazak Benkebboura, Abdelilah Iraqi Housseini, Ahmed Qaddoury

**Affiliations:** 1 Laboratory of Biotechnology, Environment, Agri-Food, and Health, Faculty of Sciences Dhar El Mahraz, Sidi Mohammed Ben Abdellah University, Fez 30050, Morocco; btissam.zoubi@usmba.ac.ma (B.Z.); abdellah.iraqihousseini@usmba.ac.ma (A.I.H.); 2 Biotechnology Unit, National Institute for Agricultural Research, INRA-Rabat, Rabat 10080, Morocco; 3 Center of Agrobiotechnology and Bioengineering, Research Unit Labeled CNRST, Cadi Ayyad University, Marrakech 40000, Morocco; c.ghoulam@gmail.com (C.G.); akachoud.oumaima@gmail.com (O.A.); abderrazakbenkebboura@gmail.com (A.B.); 4 International Maize and Wheat Improvement Center (CIMMYT), Ankara 06810, Turkey; 5 Department of Mechanical Engineering, National Yang Ming Chiao Tung University, Hsinchu 30010, Taiwan; mohammedamer.me03g@g2.nctu.edu.tw; 6 Phytopathology Unit, Department of Plant Protection, Ecole Nationale d’Agriculture de Meknès, Meknes 50001, Morocco; rlahlali@enameknes.ac.ma; 7 Laboratory of Botany, Mycology, and Environment, Faculty of Science, Mohammed V University, Rabat 10080, Morocco; laaslisalaheddine@gmail.com; 8 Research Unit on Nuclear Techniques, Environment, and Quality, National Institute for Agricultural Research, INRA-Tangier, Tangier 90010, Morocco; khalid.khfif@inra.ma; 9 Department of Plant Protection, Faculty of Agriculture, Bolu Abant Izzet Baysal University, Bolu 14030, Turkey; mustafaimren@ibu.edu.tr

**Keywords:** citrus, diversity, *Helicotylenchus* spp., nematodes, soil characteristics, *Tylenchulus semipenetrans*

## Abstract

Plant-parasitic nematodes (PPNs) are found in citrus plantations throughout the world, but they are considered to be the most problematic pest in Morocco. Citrus fruit quality and yield have been adversely affected by PPNs. Due to data unavailability of nematodes associated with citrus, a detailed survey was conducted in the main citrus-growing regions of Morocco during 2020–2021 to assess the occurrence, distribution, and diversity of PPNs associated with rhizospheres of citrus trees. In addition, some soil properties have also been assessed for their impact on soil properties. Plant-parasitic nematode diversity was calculated using two ecological indexes, the Shannon diversity index (*H′*) and the Evenness index (*E*). The collected soil and root samples were analyzed, and eleven genera and ten species of plant-parasitic nematodes were identified. The results show that the most predominant PPN species were *Tylenchulus semipenetrans* (88%), *Helicotylenchus* spp. (75%), *Pratylenchus* spp. (47%), *Tylenchus* spp. (51%), and *Xiphinema* spp. (31%). The results showed that PPN distributions were correlated with soil physicochemical properties such as soil texture, pH levels, and mineral content. Based on the obtained result, it was concluded that besides the direct effects of the host plant, physicochemical factors of the soil could greatly affect PPN communities in citrus growing orchards.

## 1. Introduction

Citrus (*Citrus* spp.) is considered one of the most extensively cultivated fruit crops worldwide, specifically in tropical and sub-tropical areas [1]. Citrus production is dominated by major producing countries including the United States, China, Brazil, Mexico, India, and Spain, with a contribution of approximately 2/3 of the global production [2]. The latter reached 147 million tons in 2017 [2]. Morocco grows citrus as one of its most important fruit crops. The sector represents an important economic element for the country, with an annual production of 1.5 to 2.0 million tons harvested from approximately 125,000 ha [1]. Additionally, the citrus industry provides an important source of foreign exchange, averaging approximately USD 0.296 billion annually [1]. The industry generates approximately 21 million jobs every year including 12 million in related orchards and nine million in packing and processing as well as many other related fields [2].

Backed by real advantages such as the Mediterranean climate, fertile soils, effective agricultural reform programs, and geographic location, Morocco is among the top ten citrus producers in the world [3]. However, the citrus sector faces several constraints that affect its production. For example, the spread of plant-parasitic nematodes (PPNs) harms citrus quality and yield [4]. Several species of PPNs have been found in the rhizosphere of citrus plants [5,6,7,8]. *Tylenchulus semipenetrans*, *Radopholus similis*, *Pratylenchus coffee*, *Longidorus*, *Hoplolaimus*, *Helicotylenchus*, *Belonolaimus longicaudatus*, and *Meloidogyne* spp. are important pests of citrus responsible for significant economic losses in several regions worldwide [9,10]. Other nematodes are considered less important pests because they rarely cause damage or are restricted to relatively small geographic areas. These include *Hemicycliophora arenaria*, *H. nudata*, *Paratrichodorus lobatus*, *P. minor*, *Pratylenchus brachyurus*, *P. vulnus*, *Xiphinema brevicolle*, and *X. index* [6].

The citrus nematode *Tylenchulus semipenetrans* is a sedentary semi-endoparasitic PPN that attacks more than 75 rutaceous species, mainly citrus [11,12]. It has been found in most citrus-growing regions of the world and in a wide variety of soil types [13,14]. Infested trees show reduced vigor, leaf chlorosis, leaf drop, dieback, and reduced fruit quality and quantity [15]. This nematode accounts for 99.1% of the total nematode community in Egypt [16], 89% in northern Iran, 26% in Florida and California (USA), and 70–90% in Spain [17]. In Morocco, *T. semipenetrans* has been found to be associated with various rootstocks in many citrus growing regions [7,18].

Nematode infection of citrus roots exacerbates under drought stress where the roots’ ability to absorb water and mineral nutrients are extremely affected [19]. In addition, root injury due to nematode penetration facilitates invasion by other pathogens such as fungi, viruses, and bacteria, which forms a disease complex [20].

Currently, there is limited information on the occurrence, density, and distribution of PPNs in citrus trees in Morocco as well as their relationship with soil physicochemical properties. Given this lack of data and information, the objectives of this study were (i) to provide more information on citrus-associated PPNs and their geographic distribution in the main citrus growing areas in Morocco, and (ii) to explore the affiliation of PPNs to soil physicochemical patterns across citrus orchards.

## 2. Materials and Methods

### 2.1. Survey and Sample Collection

**Figure 1:**
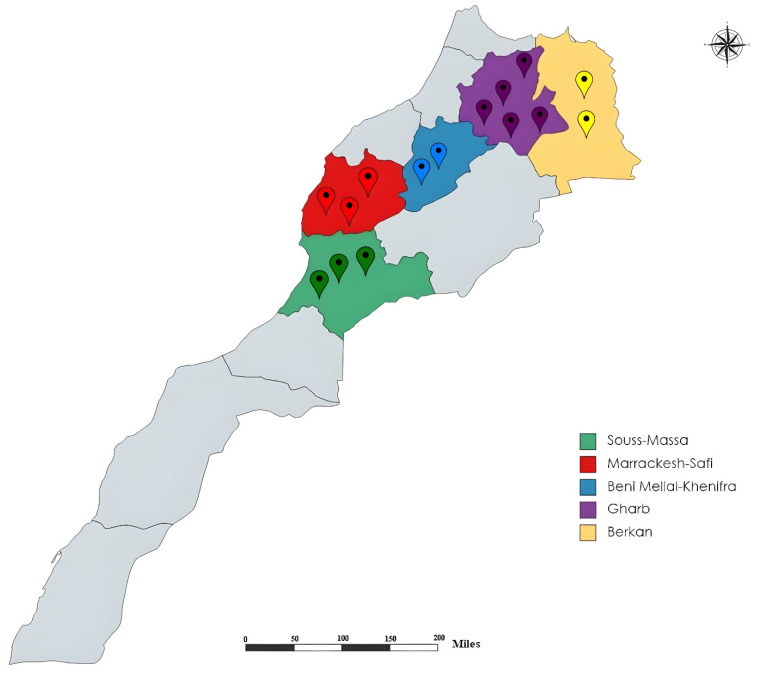
Map of the surveyed citrus-growing regions in Morocco.

Sampling was conducted during the 2020–2021 growing season in the main citrus growing areas of Morocco including Souss-Massa, Marrakech-Safi, Beni Mellal-Khenifra, Gharb, and Berkane, as shown in Figure 1. A total of 224 soil and root samples were collected from the rhizosphere of different citrus trees, as clarified in Table 1. A composite representative sample of 1 kg of soil and roots collected in a zigzag path was collected in plastic bags to avoid water loss and stored at 4 °C before analysis. Soil texture for each sampled area is summarized in Table 2. Nematological analyses were performed at the laboratory of nematology (INRA, Rabat, Morocco).

### 2.2. Nematode Extraction and Identification

Nematodes were extracted from soil and root samples using the modified Baermann technique [21]. The roots of each sample were completely cleaned in tap water and cut into small fragments (1 cm) of which 20 g was used to extract nematodes [21]. Nematodes were also extracted from each soil sample (100 g) using the same modified Baermann method. After the extraction (usually 72 h), the nematodes were stored for processing. PPN populations were expressed as the number of individuals per 100 g of soil and 20 g of root fragments. Nematodes were identified to the genus level under a stereomicroscope using reliable morphological characteristics [22,23]. The nematode samples obtained were placed in hot formalin–formaldehyde 4% [24]. The nematodes were transferred in liquid I (99 parts 4% formaldehyde + 1 part pure glycerol) to a square watch glass 7 cm in diameter. The latter was stored in a desiccator containing about one-tenths of its volume of 96% ethanol for about 12 h at 40 °C. Then, the watch glass containing the nematodes was removed from the desiccator and placed in an incubator at 37 °C. The nematodes were then prepared with the dehydration liquid II (95 parts 96% ethanol + 5 parts pure glycerol). Three ml of liquid II was added to the watch glass. The latter was partially covered with a glass slide to let it evaporate slowly. Finally, 2 mL of liquid III (50 parts 96% ethanol + 50 parts pure glycerol) was added and the watch glass was left in the incubator overnight at 37 °C. The nematodes were mounted on glass slides for light microscopic identification. *Meloidogyne* species were identified by preparing the perineal patterns [22]. Root-knot nematode (RKN) females were removed from roots and processed in a solution of sodium chloride (0.9%) for 2 min. The perineal patterns were trimmed and transferred to a drop of glycerin for microscopic examination (×100 magnification).

### 2.3. Nematode Community Assessment

Nematode diversity and incidence were determined by calculating prevalence, mean intensity, and maximum density as per the following equations [25]:

(1) $$Prevalence\;\left(\%\right)=\frac{No.\;of\;Positive\;Samples}{Total\;no.\;of\;Samples}\times 100$$

(2) $$Mean\;intensity=\frac{No.\;of\;a\;particular\;nematode\;species\;in\;the\;Positive\;Samples}{No.\;of\;Positive\;Samples}\times 100$$

(3) Maximum density=Maximum no. of a particular nematode species recovered from a sample

### 2.4. DNA Extraction, PCR, and Sequencing

For molecular identification, DNA was extracted from twenty-five individual nematodes as described by Holterman et al. [26]. The D2–D3 region was amplified with forward primers D2a (5′-ACA AGTACC GTG AGG GAA AGT TG-3′) and reverse primers D3b (5′-TCG GAA GGA ACCAGC TAC TA-3′) according to De Ley et al. [27]. One μL of DNA was added to the PCR reaction mixture containing 22 μL ddH2O, 25 μL 2 × DreamTaq PCR Master Mix (Fermentas Life Sciences, Leon-Rot, Germany), and 1 μM of the two primers. The thermocycler program consisted of 5 min at 95 °C; 35 cycles of 1 min at 94 °C, 45 s at 49 °C, and 1 min at 72 °C, followed by a final elongation step of 8 min at 72 °C. After PCR, 5 μL of each PCR product was mixed with 1 μL of 6× loading buffer (Fermentas Life Sciences, Germany) and loaded onto a 1.5% buffered standard agarose gel (TAE). After electrophoresis (100 V for 40 min), the gel was stained with ethidium bromide (0.1 μg ml^−1^) for 20 min, visualized, and photographed under UV light. The remaining PCR product was stored at −20 °C. The purified PCR products were sequenced in both directions (Macrogen) to obtain overlapping sequences of the forward and reverse DNA strands. Sequences were processed and analyzed using the Chromas 2.00 (Technelysium) and BioEdit 7.0.4.1 [28] software packages.

### 2.5. Soil Diagnosis

All analyses of the soil physicochemical properties were performed on dry and sieved (2 mm) material. Measurements of soil texture (Stxt) were determined on the proportion of clay (0–2 µm), silt (2–50 µm), and sand (50 to >200 µm) according to sedimentation estimation [29]. The pH and electrical conductivity (EC) were measured in the ratio of 1:2.5 (*w*/*v*) soil:water suspension as per the methodology described by Richards [30]. Organic matter (OM) and carbon were quantified according to the method by Anne [31]. Sodium (Na), potassium (K), and calcium (Ca) contents were determined by atomic absorption spectrometry. Total (Ptot) and available (Pass) phosphorus content was estimated using the method of Olsen et al. [32]. Total soil nitrogen (N) content was determined by the Kjeldah method [33].

### 2.6. Taxonomic Diversity

The taxonomic diversity of PPNs was assessed by calculating two nematode indices. The Shannon–Wiener index [34] is given in Equation (4):

(4) $$H^{\prime }=-\overset{s}{\underset{i-1}{\sum }}p_{i}\;Ln\;p_{i}$$

where *s* is the number of genera; *P_i_* is the proportion belonging to the corresponding number of genera; and *H′* is commonly used to characterize species diversity in a community (*H′* ranges from 0 to *Ln s*). The second index is the Evenness index [35], as given in Equation (5):

(5) $$E=\frac{H^{\prime }}{Ln\;s}$$

This quantifies the regularity of the genus distribution within the community, *E* varies between 0 and 1.

### 2.7. Data Processing

A principal component analysis (PCA) was established to define the distribution of nematode genera and the soil characteristics according to their sampling sites. Significant differences among variables were performed using Fisher’s protected least significant difference (LSD) and the Tukey test at *p* < 0.05. Differences obtained at levels of *p* < 0.05 were considered to be significant. Molecular data were processed for phylogenetic analysis. The latter relates the Moroccan nematode isolates and published sequences of each species based on the analysis of the ITS region using the maximum likelihood method and Kimura 2-parameter model [36] in MEGAX software [37]. The tree was evaluated via 1000 bootstrap replications.

## 3. Results

### 3.1. Distribution and Diversity of Plant-Parasitic Nematode Communities

**Figure 2:**
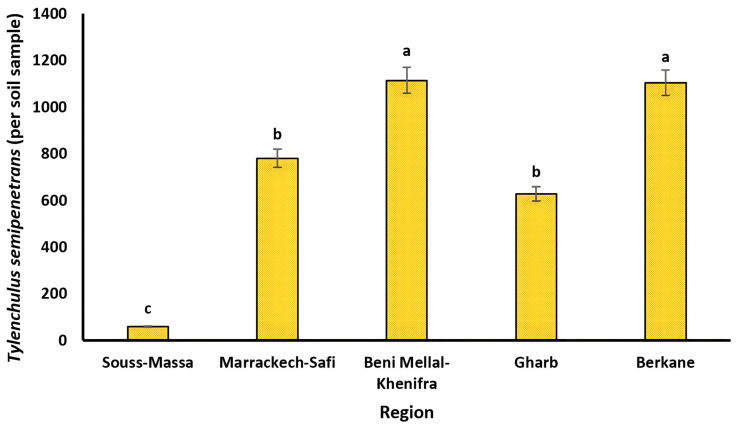
Population densities of *Tylenchulus semipenetrans* (per soil sample) in the main citrus growing regions in Morocco. Lower case letters represent the homogeneous groups based on the protected least significant difference test at *p* < 0.05.

The list of PPN species identified in all of the surveyed citrus orchards is presented in Table 3. Eleven genera and ten species of PPNs were identified morphologically from the collected soil and root samples. The data on prevalence, mean intensity, and maximum density in each studied region are presented in Table 3. Mean intensity and maximum density are quantitative parameters that are calculated to provide information about the specific proportion of plant-parasitic nematodes in soil and root matrices, and to have an idea of how far nematodes can be distributed within the plant environment, respectively. *Tylenchulus semipenetrans*, *Helicotylenchus* spp., *Pratylenchus* spp., *Paratylenchus* spp., and *Tylenchus* spp. were found in all the citrus growing regions studied. The dagger nematodes *Xiphinema diversicaudatum*, *X. americanum*, and *X. pachtaichum* were not detected in Gharb, while the root-lesion nematodes *Pratylenchus thornei* and *P. neglectus* were encountered only in the Berkane region. The major PPNs found in both soil and root samples were *T. semipenetrans* and *Helicotylenchus* spp. with high prevalence of up to 67 and 75%, respectively, while *Pratylenchus* spp. represented less than 50% of the total nematode population. Indeed, *T. semipenetrans* was highly prevalent in Marackech-Safi (62%), Beni Mellal-Khenifra (57%), Gharb (67%), and Berkane (63%), while it was least prevalent in Souss-Massa (28%). In addition, *T. semipenetrans* had the highest total density in Beni Mellal with 1114 individuals per soil sample, as shown in Figure 2.

The genera *Helicotylenchus*, *Pratylenchus*, and *Xiphinema* were found in soil and root samples with a maximum density ranging from two to 13 individuals per 100 g of soil, except in Beni Mellal-Khenifra, where *Pratylenchus* spp. had a density of 27 individuals per 100 g of soil. *Hemicycliophora* and *Criconemoides* taxa were observed in the Marrackech-Safi and Berkane regions, as they accounted for 5% of the PPNs identified. Other PPNs were also found in all regions surveyed, although in low abundance including *Tylenchorhynchus* spp., *Paratylenchus* spp., *Trichodorus* spp., *Longidorus* spp., and *Rotylenchus* spp. At species level, several nematode taxa were identified including *Pratylenchus vulnus*, *P. thornei P. neglectus*, *P. coffeae*, *Hoplolaimus indicus*, *Xiphinema pachtaichum*, *X. americanum*, and *Scutellonema bradys*.

**Figure 3:**
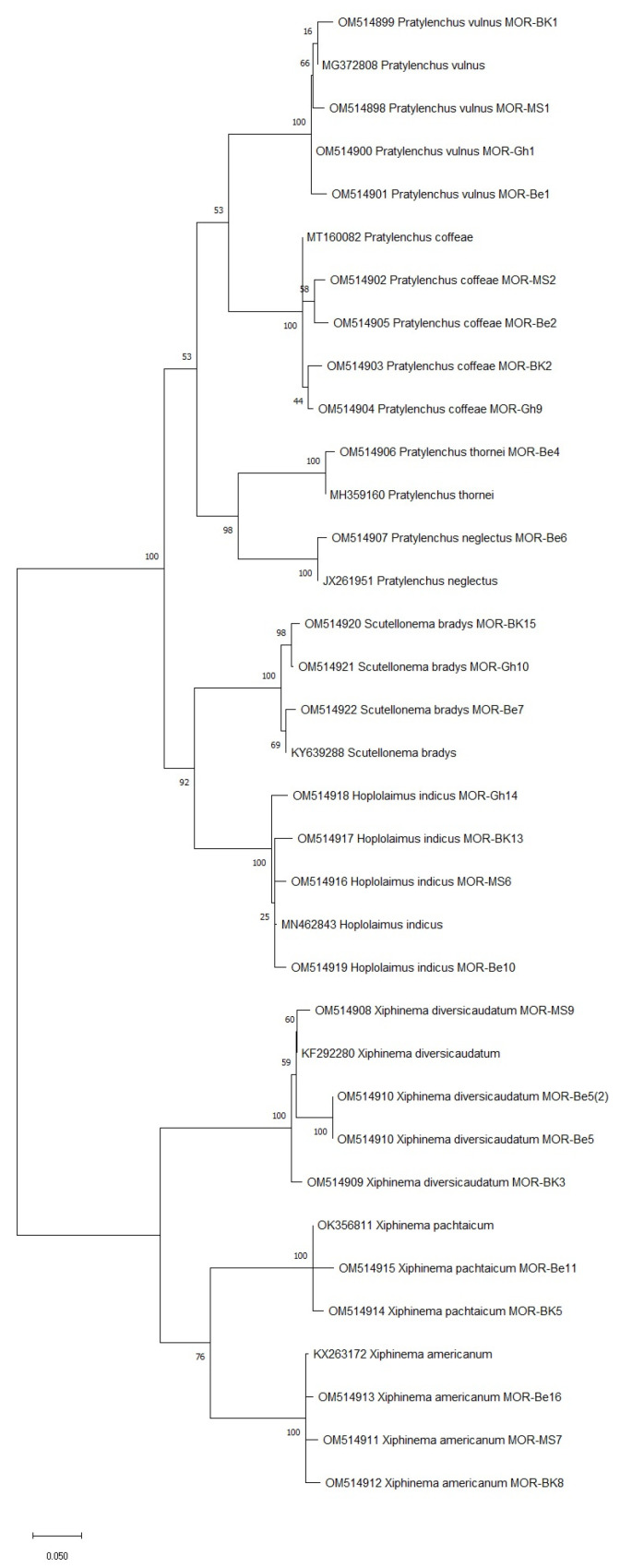
The phylogenetic tree of plant-parasitic nematode accessions detected in Moroccan citrus orchards based on the ITS region of 28S rDNA using the maximum likelihood method and Kimura 2-parameter model. The tree was generated via 1000 bootstrap replications.

To confirm the morphological identification of the PPN species isolated in this study, the D2D3 region of the 28S rDNA was sequenced. The blast test showed that the D2D3 sequences obtained matched the corresponding GenBank references by at least 99%, as presented in Table 4 and Figure 3.

The phylogenetic analysis conducted for the PPN species identified via molecular diagnosis revealed that all Moroccan species were closely related (99% similarity) to each other based on their ITS region of the 28S rDNA (Figure 3). This includes *Pratylenchus* species (*P. vulnus*, *P. coffeae*, *P. thornei*, and *P. neglectus*), *Scutellonema* (*S. bradys*), *Hoplolaimus* (*H. indicus*), and *Xiphinema* species (*X. diversicaudatum*, *X. americanum*, and *X. pachtaichum*). Interestingly, *X. americanum* is a quarantine species that was recorded for the first time in citrus orchards, which gives many implications about its damaging potential.

Based on the perennial patterns established to identify species of RKN (*Meloidogyne* spp.), *M. javanica* and *M. incognita* were found in different proportions across the surveyed citrus orchards.

**Figure 4:**
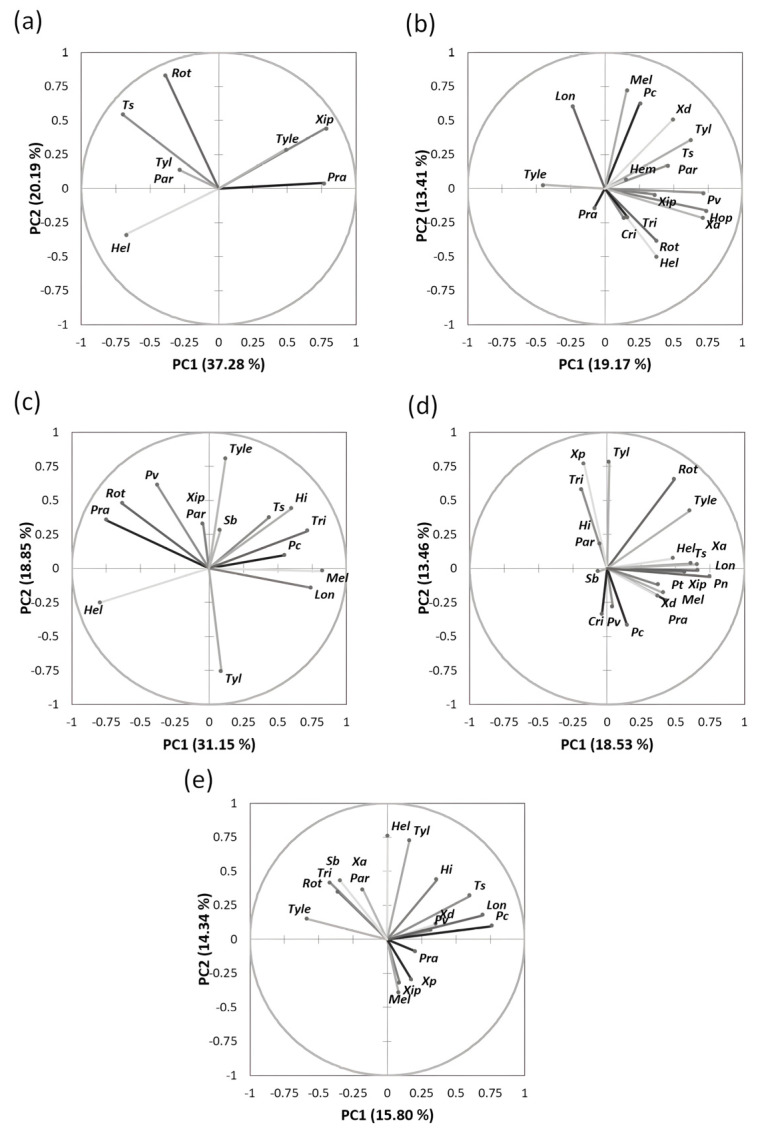
Principal component analysis of the distribution of plant-parasitic nematodes associated with citrus plants in all regions studied. (**a**) Souss-Massa region; (**b**) Marrackech- Safi region; (**c**) Gharb region; (**d**) Berkane region; (**e**) Beni Mellal-Khenifra.

The spatial distribution of the nematode genera and species in the studied regions is shown in Figure 4. For instance, the loading plot of Souss-Massa shows that the proportion of variance accounted for by the first two axes of PC was 37.28% and 20.19% (eigenvalues). The PC1 axis was associated with *Helicotylenchus* spp. (negative value). The PC2 axis was associated with *Rotylenchus* spp., *T. semipenetrans*, *Paratylenchus* spp., and *Rotylenchus* spp. (positive value). PCA plotting showed that the nematode genera had different PPN community structures in the studied citrus areas in Morocco.

### 3.2. Diversity and Community Indices

The diversity and community indices of nematodes determined in this study were evaluated using Shannon–Wiener diversity (*H′*) and evenness (*E*) indices and the number of nematode genera in each of the five citrus growing regions studied, as shown in Table 5. Significant differences (*p* < 0.05) were found among the studied citrus growing regions concerning the Shannon–Wiener index (*H′*). However, the evenness index was not significantly different among the citrus growing regions studied (*p* > 0.05). The Shannon index was higher in Marrakech-Safi (2.09), Gharb (2.05), and Beni Mellal-Khenifra (2.03) than in Berkane (1.4) and Souss-Massa (1.36). Thus, the citrus growing regions of Marrakech-Safi, Gharb, and Beni Mellal-Khenifra showed a similar trend in nematode abundance.

### 3.3. Physicochemical Properties and Their Interaction with Nematode Communities in Citrus

**Figure 5:**
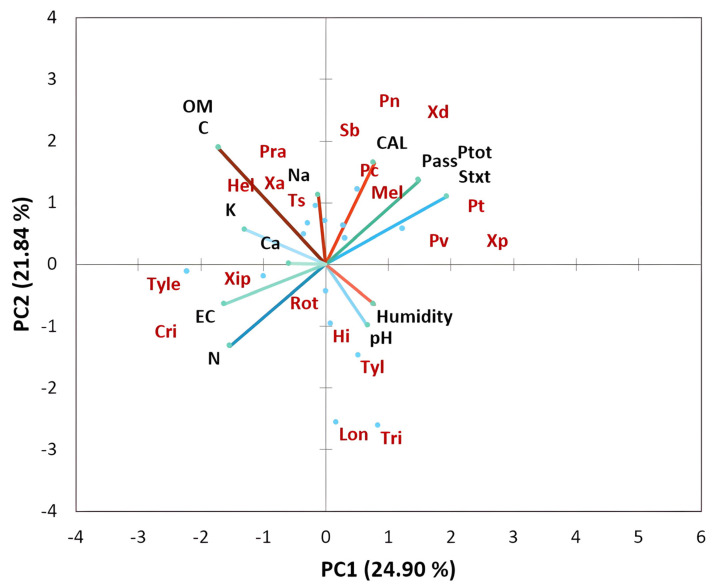
Principal component analysis (bi-plot) of soil physicochemical characteristics interacting with plant-parasitic nematode taxa associated with citrus plants in all regions studied.

The results of the principal component analysis of the physicochemical properties of the studied soils as depicted in Figure 5 show that the proportion of variance accounted for by the first two axes PC was 24.90% and 21.84% (eigenvalues), respectively. The loading plot of soil factors showed that the PC1 axis was associated with positive PC values with pH and moisture and with negative PC values with nitrogen and electrical conductivity (EC). The PC2 axis was associated with mineral content including calcium (Ca), sodium (Na), potassium (K), and organic matter (OM) in positive PC values and with EC and N in negative PC values. The bi-plot analysis of soil factors in interaction with nematode communities of citrus clearly showed that *Trichodorus* spp., *H. indicus*, *Longidorus* spp., and *Tylenchus* spp. were associated with moisture and pH. In contrast, *T. semipenetrans*, *X. americanum*, *Pratylenchus* spp., and *Helicotylenchus* spp. were particularly associated with calcium, carbon, sodium, potassium, and organic matter. However, the following genera, *Meloidogyne*, *Pratylenchus (P. thornei*, *P. coffeae*, *P. vulnus*, and *P. neglectus*), and *Xiphinema* were associated with soil texture and soil phosphorus (P) content.

## 4. Discussion

### 4.1. Distribution and Taxonomic Diversity of Plant-Parasitic Nematodes

To better understand the distribution of PPNs associated with citrus trees in Morocco, extensive surveys were conducted in the main citrus growing regions of the country. Based on the morphological and morphometric characteristics, eleven genera and ten species of plant-parasitic nematodes were identified. *Tylenchulus semipenetrans* was the most common PPN in the citrus orchards surveyed, as reported in other citrus growing areas in Morocco [7] and worldwide including Iran [38], Egypt [39,40], and Spain [12]. Indeed, *T. semipenetrans* was the predominant nematode found in Souss-Massa (88%), Marackech-Safi (62%), Beni Mellal-Khenifra (57%), Gharb (67%), and Berkane (63%). This species is notable not only for its close association with citrus, but also for the ability of citrus trees to support very high populations before vigor declines and symptoms appear. The wide distribution of *T. semipenetrans* could be attributed to many factors including infected seedlings, contaminated plant material, organic fertilizers, irrigation, and machinery [39]. In addition, the high variability of PPN genera observed in this study can be attributed to the variation in ecological and edaphic factors between and within the different regions studied [41].

The second most prevalent plant-parasitic nematode was *Helicotylenchus* spp., which varied from 75% in Souss-Massa to 38% in Beni Mellal-Kenitra and Gharb. The prevalence of this genus in Moroccan citrus-growing regions was higher than that in Spain [12] and Egypt [40]. On the other hand, a higher prevalence of *H. dihystera* (80%) was reported by Kumar and Das in Tinsukia [42]. Four species of *Pratylenchus* spp. (*P. vulnus*, *P. thornei*, *P. neglectus*, *P. coffeae*) were identified in the surveyed regions. Population densities of this genus ranged from 27 to six nematodes per 100 g soil in Beni Mellal-Khenitra and Gharb, respectively, and from 17 to three nematodes per 20 g roots in Berkane and Souss-Massa, respectively. Several studies have reported the presence of these nematode species in citrus orchards worldwide. In Brazil, *P. coffeae* infests about 1% of citrus nurseries and orchards [43], while it has a major trend in Florida, USA [44]. Other RLN species were described to be prominent such as *P. vulnus* and *P. neglectus* in Israel [45], and *P. vulnus* and *P. coffeae* in Morocco [7]. These species can significantly reduce root weight, which translated into significant yield losses [4]. These nematodes have been reported as one of the main pests limiting raspberry and saffron production in Morocco [46,47]. The dagger nematode *Xiphinema* spp. is among the ten most economically important PPNs [48]. This genus causes serious problems for organic farming in Egypt [49], raspberry and citrus in Morocco [46,50], and vegetable production in Saudi Arabia [51]. This migratory ectoparasitic nematode is particularly problematic because it can harbor and transmit plant viruses. Thus, even at low densities, these nematode species can be very damaging to plants [52,53].

The distribution of *Xiphinema* species identified in this study varied considerably among the citrus-growing regions. *X. pachtaichum* was observed only in Beni Mellal-Khenifra (3.3%) and Berkane (3.7%), *X. americanum* was found in Marackech-Safi (12%), Beni Mellal-Khenifra (6.7%), and Berkane (1.9%), while *X. diversicaudatum*, which was first reported in Morocco in May 2012 in the Gharb region, was encountered in Marackech-Safi (6.6%), Beni Mellal-Khenifra (10%), and Berkane (7.4%). According to Mokrini et al. [50], *X. diversicaudatum* was first reported in Moroccan citrus orchards. This nematode can transmit the Arabis mosaic virus, mainly associated with grapevine fanleaf degeneration disease [50]. On the other hand, *X. americanum* was reported for the first time ever, associated with citrus in Morocco. This is extremely interesting, giving the economic importance and quarantine aspect of this nematode and its potential damage attributed to citrus crops.

The root-knot nematode *Meloidogyne* spp., attacking citrus crops was not frequently reported due to its limited distribution. In the present study, only a few citrus-growing fields were found to be infested by these nematodes, and had prevalence values of 23, 25, 22, and 13% in Marackech-Safi, Beni Mellal-Khenifra, Gharb, and Berkane, respectively. This prevalence of *Meloidogyne* spp. was lower than that in Egyptian citrus orchards intercropped with tomato [8], which implies the decent linkage between these nematodes and citrus crops rather than its presence in intercropped trials with native hosts or in weeds. Several studies recorded in Taiwan and India have reported that *Meloidogyne* spp. could cause elongated galls on citrus roots [11]. Moreover, Some RKN species (e.g., *M. incognita*, *M. javanica*, and *M. arenaria*) were found in the infection zones of Troyer citrange and sour orange rhizospheres, causing small galls without reproduction activity [54]. Many of the nematode genera associated with citrus in the present study have also been previously reported for the same crop worldwide. The needle nematode *Longidorus* spp., the stubby root nematode *Trichodorus* spp., the stunt nematode *Tylenchorhynchus* spp., the ring nematode *Criconemoides*, and the spiral nematode *Rotylenchus* spp. are commonly found in the surveyed citrus growing areas. Indeed, *Tylenchorhynchus* spp. and *Longidorus* spp. have been detected in Egypt [8], Spain [12], and Tinsukia [42]. In our study, *S. bradys* and *H. indicus* were found at low densities that varied between two and four nematodes per 100 g of soil and between two and seven nematodes per 100 g of soil, respectively. According to Kumar and Das [42], *H. indicus* was very abundant in citrus orchards in Tinsukia. The sheath nematode *Hemicycliophora* spp. was the least abundant nematode. It was observed in 5% of the sampled citrus plots in the Marackech-Safi region with an average density of two nematodes per 100 g of soil. These results are in agreement with the scientific work of Shanmugam et al. [55], who found that *Hemicycliophora* spp. was least prevalent in some Indian weeds, shrubs, and herbs. In addition, it has been reported that *H. ahvasiensis* was isolated from the soil and root matrices of citrus in Egypt. However, the damage amplitude caused by this nematode to the citrus trees have not been documented yet [4]. Therefore, the rare abundance of this nematode could probably be related to the soil’s temperature, irrigation, and aeration, alongside the seasonal attributes [4,17].

The results of the analysis of the Shannon (*H′*) and evenness (*E*) diversity indices showed that *H′* values were highest in Marrakech-Safi, Gharb, and Beni Mellal-Khenifra, which have extended dry seasons. Freitas et al. [56] indicated that dry season and soil depth (0–10 cm) favored the total population of PPNs associated with citrus plants in Brazil.

### 4.2. Relationship between Plant-Parasitic Nematode Communities and Soil Physicochemical Factors

Understanding the interaction between soil physicochemical properties and plant-parasitic nematodes is critical for effective and environmentally-friendly management. The citrus nematode, *T. semipenetrans* was positively correlated with soil mineral nutrients (K, Ca, Na, and C) and organic matter content. A strong correlation was found between these parameters and the prevalence of *T. semipenetrans* in citrus growing areas in Spain [12]. In contrast, Benjlil et al. [57] reported a negative correlation between the prevalence of PPNs parasitizing saffron and soil organic matter. Soil organic matter accumulation could significantly reduce PPN abundance in wheat via decreasing their vital proprieties [58]. Moreover, the prevalence of *Pratylenchus* spp., *Helicotylenchus* spp., and *X. americanum* was closely related to the mineral content including Fe, Ca, and Na [59,60]. In addition, Francl [61] observed that the population density of *Heterodera glycines* was positively correlated with magnesium (Mg) content. In this study, most of the identified PPNs showed a negative correlation with nitrogen (N), except for *Criconemoides*, *Rotylenchus* spp., and *Tylenchorhynchus* spp. Interestingly, the accumulation of nitrate via nitrification is considered destructive to PPNs [62]. Phosphorus content was positively correlated with the occurrence of *Meloidogyne* spp., *Xiphinema* spp., *P. thornei*, *P. vulnus*, and *P. neglectus*. Nisa et al. [41] reported the same positive correlation between nematode abundance and soil P content. Soil pH also had a strong influence on the abundance of citrus nematodes. Low pH increased nematode abundance and diversity [63,64]. Soils with acidic pH increase the proliferation of the root-knot nematode *Meloidogyne* spp. [65,66,67]. In contrast, Salahi Ardakani et al. [68] found that the highest abundance of *T. semipenetrans* was observed in soils of pH 7. Moreover, Van Gundy and Martin [69] indicated that the density of *T. semipenetrans* in citrus was four times higher in neutral soils than in acidic soils.

Soil texture and structure significantly affect the movement of soil nematodes. Our results indicated that the texture of fine sandy soil positively affects the distribution of nematodes. Salahi Ardakani et al. [68] found that the maximum abundance of the citrus nematode *T. semipenetrans* was found in clay soils. A recent study by Laasli et al. [70] showed that *Aphelenchoides* spp., *Merlinius* spp., and *Pratylenchus* spp. were associated with sandy and silt soils in wheat fields. In contrast, *Longidorus* spp., and *Xiphinema* spp. were more common on soils with higher clay content. Mokrini et al. [46] found that the abundance of several plant-parasitic nematodes affecting raspberries was strongly associated with soil granulometry. Kim et al. [71] indicated that sandy soils favor the growth of nematodes such as *M. incognita* by promoting their motility and feeding activities.

## 5. Conclusions

This study highlights the main plant-parasitic nematode diversity found in the Moroccan citrus-growing areas. The citrus nematode *T. semipenetrans* was the most abundant nematode identified in the soil and root matrices. This is probably because the local environmental and soil conditions are more suitable for its growth. Other economically important nematode species (e.g., *P. vulnus*, *P. thornei*, *S. bradys*, *H. indicus*, and *X.* *diversicaudatum*) were recorded as well as *X. Americanum*, which has been reported for the first time in citrus orchards. The relationship of these nematodes with edaphic proprieties has been revealed in the sense that it may help farmers to accurately tackle problematic PPNs. Additionally, the results of this study will be of great value to researchers and pest management authorities to control and reduce the spread of PPNs to improve citrus production in Morocco.

## Figures and Tables

**Table 1:** Distribution of samples analyzed by prospecting area and citrus rootstocks.

Prospecting Area (Region)	Number of Samples	Rootstock Variety
Souss-Massa	32	Carrizo citrange
		Sour orange
		macrophylla
Marrackech-Safi	60	Sour orange
		‘Australian’ sour orange
		Carrizo citrange
		Volkameriana
		C-35 citranges
Beni Mellal-Khenifra	60	Sour orange
		Carrizo citrange
		Volkameriana
		C-35 citranges
		‘Australian’ sour orange
Gharb	18	Troyer citrange
		Sour orange
		Carrizo citrange
Berkane	54	Sour orange
		Carrizo citrange
		‘Australian’ sour orange
Total	224	

**Table 2:** Texture and pH of the soils of the sampled citrus orchards.

Region	Orchard	Soil Texture	Soil pH
Souss-Massa	Balfa	Sandy loam	7.65
	Taroudant	Sandy loam	8.32
	Biogra	Sandy clay loam	7.86
Marrackech-Safi	Souihla	Sandy loam	8.57
	Es Saada	Sandy loam	8.27
	Agafai	Sandy loam	7.42
Beni Mellal-Khenifra	Souk Sebt	Silty clay	7.89
	Guettaya	Silty clay	7.03
Gharb	M’nasra	Sandy	7.43
	Allal Tazi	Sandy	8.05
	Ouled Azouz	Sandy loam	7.64
	Sidi Slimane	Sandy clay loam	7.81
	Sidi Kacem	Sandy loam	8.23
Berkane	Zegzel	Sandy clay	8.13
	Aglmine	Sandy loam	7.59

**Table 3:** Prevalence, mean intensity, and the maximum density of plant-parasitic nematodes from soil (100 g) and root (20 g) of citrus in the main citrus growing areas of Morocco.

Nematode Taxa/Genus and Species	Souss-Massa					Marrackech-Safi					Beni Mellal-Khenifra					Gharb					Berkane				
	Pr	Intensity		Density		Pr	Intensity		Density		Pr	Intensity		Density		Pr	Intensity		Density		Pr	Intensity		Density	
		Root	Soil	Root	Soil		Root	Soil	Root	Soil		Root	Soil	Root	Soil		Root	Soil	Root	Soil		Root	Soil	Root	Soil
*T. semipenetrans* (*Ts*)	28	2	4	5	17	62	11	34	36	121	57	25	49	58	169	67	14	90	34	203	63	14	156	27	213
*Pratylenchus* spp. (*Pra*) *	47	2	3	3	8	50	3	4	11	7	43	6	8	16	27	39	3	4	4	6	44.4	6	3	17	8
*P. vulnus (Pv)*	-	-	-	-	-	15	2	3	4	5	18	5	4	2	5	17	3	2	3	2	13	3	3	4	4
*P. coffeae (Pc)*	-	-	-	-	-	20	4	3	4	6	20	4	4	5	5	18	1	3	1	4	18.8	5	3	5	5
*P. thornei (Pt)*	-	-	-	-	-	-	-	-	-	-	-	-	-	-	-	-	-	-	-	-	1.9	-	3	-	3
*P. neglectus (Pn)*	-	-	-	-	-	-	-	-	-	-	-	-	-	-	-	-	-	-	-	-	3.7	-	5	-	5
*Paratylenchus* spp. (*Par*)	16	2	3	2	3	15	2	4	4	5	22	4	3	5	5	11	3	4	4	4	14.8	3	2	3	3
*Helicotylenchus* spp. (*Hel*)	75	1	3	2	5	60	3	5	9	13	38	5	7	11	11	39	2	4	2	7	46.3	4	6	11	14
*Tylenchus* spp. (*Tyl*)	25	3	5	4	7	52	-	11	-	15	28	4	68	5	15	33	12	9	12	11	24	5	9	8	17
*Xiphinema* spp. (*Xip*) *	31	0	2	0	2	30	-	7	-	11	22	5	8	-	13	28	3	2	3	3	16.7	-	5	-	11
*X. diversicaudatum (Xd)*	-	-	-	-	-	6.6	-	3	-	4	10	-	4	-	9	-	-	-	-	-	7.4	-	3	-	3
*X. americanum (Xa)*	-	-	-	-	-	12	-	3	-	5	6.7	-	2	-	3	-	-	-	-	-	1.9	-	3	-	1
*X. pachtaichum (Xp)*	-	-	-	-	-	-	-	-	-	0	3.3	-	2	-	2	-	-	-	-	-	3.7	-	5	-	2
*Hoplolaimus indicus (Hi)*	-	-	-	-	-	5	-	2	-	3	10	-	3	-	6	11	-	4	-	5	5.5	-	3	-	4
*Scutellonema bradys (Sb)*	-	-	-	-	-	-	-	-	-	0	1.7	-	7	-	7	5.5	-	3	-	3	1.9	-	2	-	3
*Rotylenchus* spp. (*Rot*)	25	2	0	0	3	8.3	3	-	-	4	15	3	4	3	7	5.5	-	3	-	3	11.1	-	4	-	7
*Tylenchorhynchus* spp. (*Tyle*)	19	0	2	0	3	13	-	5	-	9	15	-	11	0	8	17	-	2	-	2	16.7	-	6	-	12
*Meloidogyne* spp. (*Mel*)	-	-	-	-	-	23	4	3	5	6	25	4	5	7	12	22	3	2	3	3	13	2	3	2	5
*Trichodorus* spp. (*Tri*)	-	-	-	-	-	8.3	-	2	-	4	6.7	-	4	-	4	11	-	2	-	2	3.7	-	3	-	2
*Criconemoides (Cri)*	-	-	-	-	-	5	-	3	-	4	-	-	-	-	-	-	-	-	-	-	1.9	-	2	-	-
*Hemicycliophora* spp. (*Hem*)	-	-	-	-	-	5	-	2	-	2	-	-	-	-	-	-	-	-	-	-	-	-	-	-	-
*Longidorus* spp. (*Lon*)	-	-	-	-	-	13	-	2	-	2	18	7	4	7	7	11	-	3	-	3	11.1	-	-	-	-

- Absence of nematodes; Pr: Prevalence (accounted for both juvenile and adult developmental stages of nematodes); * Assessment includes non-identified juveniles.

**Table 4:** Molecular diagnosis of the main plant-parasitic nematode species in citrus, based on the 28S rDNA region, with their Genbank accession codes.

Nematode Species	Region	Collection Codes for DNA Sequences	Genbank Accession Codes
*Pratylenchus vulnus*	ITS	MOR-Be1-Pratylenchus MOR-MS1-Pratylenchus MOR-BK1-Pratylenchus MOR-Gh1-Pratylenchus	OM514901 OM514898 OM514899 OM514900
*Pratylenchus coffeae*	ITS	MOR-MS2-Pratylenchus MOR-BK2-Pratylenchus MOR-Gh9-Pratylenchus MOR-Be2-Pratylenchus	OM514902 OM514903 OM514904 OM514905
*Pratylenchus thornei*	ITS	MOR-Be4-Pratylenchus	OM514906
*Pratylenchus neglectus*	ITS	MOR-Be6-Pratylenchus	OM514907
*Scutellonema bradys*	ITS	MOR-BK15-Scutellonema MOR-Gh10-Scutellonema MOR-Be7-Scutellonema	OM514920 OM514921 OM514922
*Hoplolaimus indicus*	ITS	MOR-MS6-Hoplolaimus MOR-BK13-Hoplolaimus MOR-Gh14-Hoplolaimus MOR-Be10-Hoplolaimus	OM514916 OM514917 OM514918 OM514919
*Xiphinema diversicaudatum*	ITS	MOR-MS9-Xiphinema MOR-BK3-Xiphinema MOR-Be5-Xiphinema	OM514908 OM514909 OM514910
*Xiphinema americanum*	ITS	MOR-MS7-Xiphinema MOR-BK8-Xiphinema MOR-Be16-Xiphinema	OM514911 OM514912 OM514913
*Xiphinema pachtaichum*	ITS	MOR-BK5-Xiphinema MOR-Be11-Xiphinema	OM514914 OM514915

**Table 5:** Shannon–Wiener diversity (*H′*), evenness (*E*), and the number of nematode genera in each of the five surveyed citrus regions.

Regions	Diversity Parameters		
	Number of PPNs *	Shannon Diversity Index (*H′*) *	Evenness (*E*) *
Souss-Massa	2.91 c	1.36 b	0.7 a
Marrakech-Safi	12.65 b	2.09 a	0.84 a
Beni Mellal-Khenifra	18.05 a	2.03 a	0.88 a
Gharb	3.3 c	2.05 a	0.89 a
Berkane	3.69 c	1.4 b	0.84 a
*p*	<0.05	<0.05	>0.05

* Numbers in the same column followed by different letters were significantly different based on Tukey’s test.

## Data Availability

Not applicable.
